# Medical Journalism

**Published:** 1860-07

**Authors:** 


### Medical Journalism.

Such as have not reveled in the easy luxuriance of an editorial chair, have much of the delectable in life to realize, and many of the pleasures arising from proofs of appreciated services to enjoy. The busy whirl of professional life, the intensity of its daily cares, the frequency of its vigils, ingratitude in the recipients of its blessings, the inadequacy of its pecuniary returns, and a thousand other annoying attendants upon its active pursuit, sink into a hushed oblivion, and in their stead appear the pleasing consciousness of services remunerated, grateful smiles of delighted readers, and the beneficent countenances of promptly paid publishers, as quietly in the still hours of night, your rapid pen records the thoughts which an anxious multitude of readers are to profit by. Such, doubtless, is the reasoning of those who do not desecrate the sacredness of our Elysium by intimations of our supposed mortality or necessities.

Thus argue those who do not deem us made of such rude stuff as dust and ashes. So they congratulate themselves, who do not care to pay the printer. But, kind reader, while you exact the performance of certain duties from us, and in a spirit of hypercriticism examine and decide upon their performance, remember that you also have duties to perform, and the first should be to pay the printer. If what you receive is not an equivalent for the picayune expenditure, then refuse to continue as a subscriber, first, however, having determined whether the deficiency arises from your own imbecility or the incompetency of the editors. Such as are at all interested in our success will please manifest that interest, by paying what they owe. More, we do not require—less will be insufficient. On the first of July we hope to balance our books, and disprove the assertions of those who say that there is not energy or public spirit enough in the South-west to sustain a medical journal.—*Louisville Medical News.*

